# Cytotoxic effects of cobalt and nickel ions on osteocytes *in vitro*

**DOI:** 10.1186/s13018-014-0091-6

**Published:** 2014-10-08

**Authors:** Arihiko Kanaji, Vbenosawemwinghaye Orhue, Marco S Caicedo, Amarjit S Virdi, Dale R Sumner, Nadim J Hallab, Toyama Yoshiaki, Kotaro Sena

**Affiliations:** Department of Anatomy & Cell Biology, Rush University Medical Center, 600 South Paulina Street, Armour Academic Center Suite 507, Chicago, IL 60612 USA; Department of Orthopedic Surgery, Rush University Medical Center, 1725 West Harrison Street, Cohn Research Building Suite 722, Chicago, IL 60612 USA; Department of Orthopaedic Surgery, Keio University, School of Medicine, Tokyo, 160-8582 Japan; Department of Periodontology, Kagoshima University, Kagoshima, 890-8544 Japan

**Keywords:** Metal-on-metal bearing surfaces, Cobalt-chromium-molybdenum, Metal ions, Osteocytes

## Abstract

**Background:**

Metal-on-metal prostheses undergo wear and corrosion, releasing soluble ions and wear particles into the surrounding environment. Reports described early failures of the metal-on-metal prostheses, with histologic features similar to a Type IV immune response. Mechanisms by which metal wear products and metal ion causing this reaction are not completely understood, and the effects of metal ions on osteocytes, which represent more than 95% of all the bone cells, have not been also studied. We hypothesized that soluble metal ions released from the cobalt-chromium-molybdenum (Co-Cr-Mo) prosthesis may have cytotoxic effect on osteocytes.

**Methods:**

MLO-Y4 osteocytes were treated with various metal ion solutions for 24 and 48 h. The effect of ion treatment on cytotoxicity was assessed by WST-1 reagents and cell death ELISA. Morphological changes were analyzed by a phase-contrast microscope or fluorescent microscope using Hoechst 33342 and propidium iodine staining.

**Results:**

Cr and Mo ions did not cause cell death under 0.50 mM, highest concentration studied, whereas Co and Ni ions had significant cytotoxic effect on MLO-Y4 cells at concentrations grater than 0.10 mM and at 0.50 mM, respectively, in a dose-dependent manner. According to the ELISA data, osteocytes treated with Co ions were more susceptible to necrotic than apoptotic cell death, while Ni ions caused osteocyte apoptosis. The morphological assays show that cells treated with Co and Ni ions at high concentration were fewer in number and rounded. In addition, fluorescent images showed a marked reduction in live cells and an increase in dead osteocytes treated with Co and Ni ions at high concentration.

**Conclusions:**

Metal ions released from metal-on-metal bearing surfaces have potentially cytotoxic effects on MLO-Y4 osteocytes, *in vitro*.

## Background

In 2005, approximately 250,000 total hip arthroplasties were performed in the United States [1], and according to a recent report, more than 30% had metal-on-metal bearings [2]. The use of a metal-on-metal bearing in total hip arthroplasty and hip resurfacing arthroplasty is advantageous in terms of lower volumetric wear rate (by 20 to 100 times) in comparison to conventional metal-on-polyethylene bearings [3]. Other benefits include high fracture toughness and the ability to use large femoral heads, which reduces the risk of postoperative instability [4]. However, it has been well documented that metal-on-metal prostheses in contact with host tissue undergo wear and corrosion, releasing soluble ions and wear particles into the surrounding environment [4]. Elevated levels of metal ions, such as cobalt and chromium, have been detected in the body fluids; serum, whole blood, synovial fluid, and tissues obtained from subjects with well-functioning metal-on-metal implants [5–8]. It is unclear to what extent exposure to soluble metal ions released from implants over the long run can lead to; it may lead to a poor outcome such as aseptic loosening. The high usage (44.8%) of metal-on-metal bearing surfaces in patients below 55 years old [2] has raised concerns about the long-term biological effects of metals [9], including toxicity and sensitivity reactions.

Aseptic loosening is mostly attributed to wear debris particles. Cells of the monocyte/macrophage lineage play a primary role by phagocytosing wear particles that lead to the activation and release of pro-inflammatory mediators which accelerate bone resorption around the implant [10]. While other cell types such as lymphocytes, endothelial cells, fibroblasts, bone marrow-derived mesenchymal cells, osteoblasts, and osteoclasts contribute to the pathology, according to numerous studies [10], osteocytes, which represent more than 95% of all the bone cells, have not been examined. Moreover, the precise etiology of aseptic loosening still remains unclear.

Osteocytes are terminally differentiated cells of the osteoblast lineage that have become embedded in a mineralized bone matrix and may send signals that regulate bone modeling and remodeling [11]. Although the role of osteocytes remained unknown for a long time, numerous reports highlight their role by demonstrating the linkage between osteocyte death/apoptosis to the control of local bone resorption [12–16]. More recently, it has been suggested that osteocytes may be involved in the inflammatory response and subsequent periprosthetic bone resorption following total joint arthroplasty [17–19]. Metal (cobalt-chromium-molybdenum; Co-Cr-Mo) wear particles were able to stimulate production of the pro-inflammatory cytokine by osteocytes that could lead to osteocyte apoptosis, *in vitro* [18]. However, the effects of soluble metal ions on osteocytes have not been studied. We hypothesized that soluble metal ions released from Co-Cr-Mo prostheses will have a cytotoxic effect on osteocytes at clinically relevant concentrations [5–8,20–22], suggesting a role of osteocytes in the pathophysiology of implant failure.

## Materials and methods

### Cell culture

MLO-Y4, a murine long bone-derived osteocytic cell line, derived from 14-day-old transgenic mice containing the SV40 large T-antigen driven by the osteocalcin promoter 23] was kindly provided by Dr. Lynda F. Bonewald (University of Missouri-Kansas City, Kansas City, MO, USA) for use in this study. The MLO-Y4 cell line displays an osteocyte phenotype. Cells were cultured at 37°C, 5% CO_2_, 95% air in α-MEM supplemented with 2.5% fetal bovine serum, 2.5% calf serum, and antibiotics on tissue culture plastic dishes coated with rat tail collagen (BD Biosciences, San Jose, CA, USA) as previously described [23]. Cells were plated at a density of 1.0 × 10^5^ cells/well in 12-well culture plates.

### Soluble metal solutions

After a 24-hr pre-culture period, the MLO-Y4 cells were treated with four concentrations of sodium (Na^+^), cobalt (Co^2+^), chromium (Cr^3+^), molybdenum (Mo^5+^), and nickel (Ni^2+^) chloride solutions at 0.00 (control), 0.05, 0.10, and 0.50 mM, respectively. These concentrations were chosen based upon a previous study utilizing osteoblasts in which Co and Ni at 0.50 mM (clinically relevant concentrations) demonstrated cell toxicity [22]. Except for Na which was used for comparison, all the metals tested were components of Co-Cr-Mo implant alloys. Stock metal ion solutions (10 mM) were prepared by dissolving sodium (NaCl), cobalt (CoCl2), chromium (CrCl3), molybdenum (MoCl5), and nickel (NiCl2) chloride powders (Sigma-Aldrich, St. Louis, MO, USA) with double-deionized water (Milli-Q water system; Millipore, Bedford, MA, USA), filter-sterilized before use as described previously [22] and added to cell cultures to the final concentrations listed above.

### Cytotoxicity assay

Cell proliferation reagent WST-1 (Roche Diagnostics, Indianapolis, IN, USA) was used to investigate the cytotoxic effect of the metal ions after 48 hr. In brief, after a 48-hr metal ion treatment, a ready-to-use WST-1 solution was added and incubation continued for 2 hr at 37°C. The reaction product was measured at 450 nm with the reference wavelength at 630 nm by a microplate reader (BioTek, Winooski, VT, USA).

### Detection of apoptosis and necrosis

The Cell Death Detection ELISA^PLUS^ photometric enzyme immunoassay kit (Roche Diagnostics, Indianapolis, IN, USA) was used to analyze the apoptotic and necrotic cell death quantitatively after 24 hr. The kit utilizes two monoclonal antibodies directed against DNA and histones, respectively, to allow the specific determination of mono- and oligo-nucleosomes in the cytoplasmatic fraction (apoptosis) or in the supernatant (necrosis) of cells.

### Cell morphology

Morphological analyses were done under a phase-contrast microscope (Nikon, Tokyo, Japan) and fluorescent microscope (Nikon, Tokyo, Japan) using propidium iodide (Sigma-Aldrich, St. Louis, MO, USA) and Hoechst 33342 (Biotium, Hayward, CA, USA). The propidium iodide was used to identify dead cells while Hoechst 33342 was used to stain the total (dead and healthy) cells. The charged dye propidium iodide is excluded by cells with intact membranes, whereas Hoechst 33342 stains DNA in all cells. In brief, MLO-Y4 cells were washed once in PBS and incubated in a staining solution which contains propidium iodide (10 μM) and Hoechst 33342 (25 μM) at room temperature for 15 min. Cells were washed with PBS and then analyzed by fluorescence microscopy using filters for rhodamine (red) and 4′,6-diamidino-2-phenylindole dihydrochloride (DAPI; blue).

### Statistical analysis

For quantitative results, all values are presented as mean ± standard deviation of at least four replicates. These values were analyzed with two-way ANOVA and the Bonferroni/Dunn test as a *post hoc* test for all groups using GraphPad Instat® software (GraphPad Software Inc., San Diego, CA, USA). Significance was defined as probability values less than 0.05 (*p* <0.05).

### Source of funding

This study was supported by a grant from NIH (R21AR054171 and T32AR052272), the Rush University Committee on Research grant, and the Grainger Foundation.

## Results

### Cytotoxicity assay

**Figure 1:**
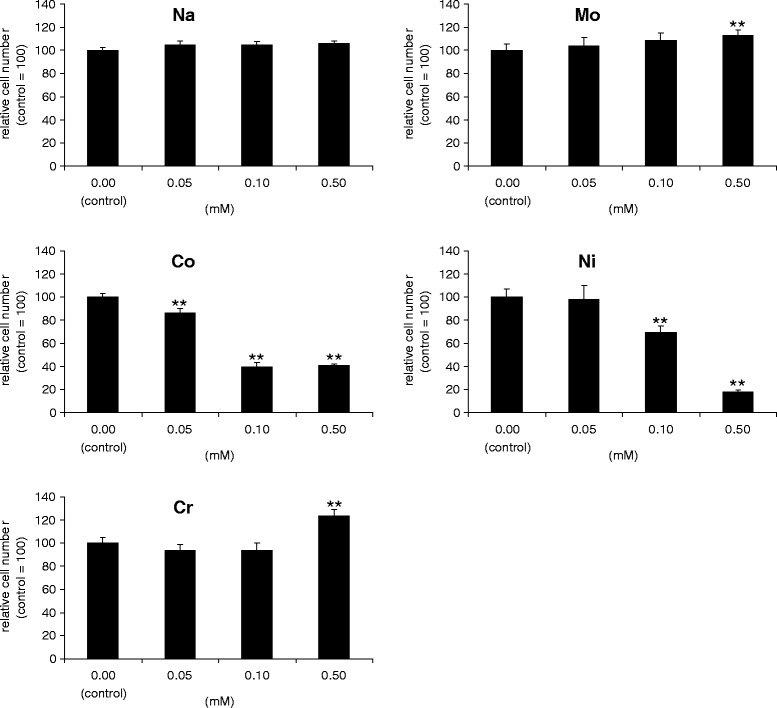
**Cytotoxic effects of metal ion solution on MLO-Y4 osteocytes.** Cytotoxicity of Na, Co, Cr, Mo, and Ni ions was analyzed by a WST-1 reagent after 48 hr. Cytotoxicity is demonstrated as relative to 0.00 (control). Mean values ± SD (*n* =6) are represented. The asterisk indicates statistically significant difference (**p <0.01 vs. control).

A cytotoxicity assay was performed to quantify the cytotoxic effect of soluble metal ions at 48 hr. Among the four metal ion solutions studied, Co and Ni ions had significant cytotoxic effect on MLO-Y4 cells (Figure 1). Co ions had a significant cytotoxic effect at concentrations above 0.05 mM (*p* <0.01). After 48 hr, MLO-Y4 cells in 0.05, 0.10, and 0.50 mM Co-ion-treated groups were 86.3% ±3.6%, 39.7% ±3.5%, and 40.7% ± 1.5% of control values, respectively. Ni ions had a significant cytotoxic effect at 0.10 and 0.50 mM which was 69.8% ±4.9% and 17.9% ±1.6% of control (*p* <0.01) while Cr and Mo ions had a proliferative effect at 0.50 mM which was 123.7% ±5.4% and 110.0% ±2.1% of control (*p* <0.05). Na ions which were used for comparison did not show significant differences at any concentrations studied.

### Detection of apoptosis and necrosis

**Figure 2:**
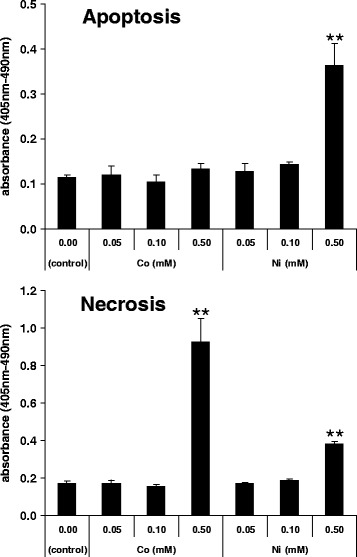
**Analysis of cell death (apoptosis/necrosis) by cell death ELISA.** For Co- and Ni-ion-treated groups, cytoplasmatic fraction and the supernatant of MLO-Y4 were collected to measure apoptosis and necrosis levels after 24 hr. Mean values ± SD (*n* =6) are represented. The asterisk indicates statistically significant difference (**p <0.01 vs. control).

The Cell Death Detection ELISA^PLUS^ photometric enzyme immunoassay was used to characterize cell death induced by Co and Ni ions at 24 hr. Results from the immunoassay indicated significantly high levels of necrosis after Co ion treatment at 0.50 mM (*p* <0.01), compared to control. Significantly higher levels of both apoptosis and necrosis were observed for MLO-Y4 cells treated with 0.50 mM Ni ion solution (*p* <0.01) (Figure 2).

### Cell morphology

**Figure 3:**
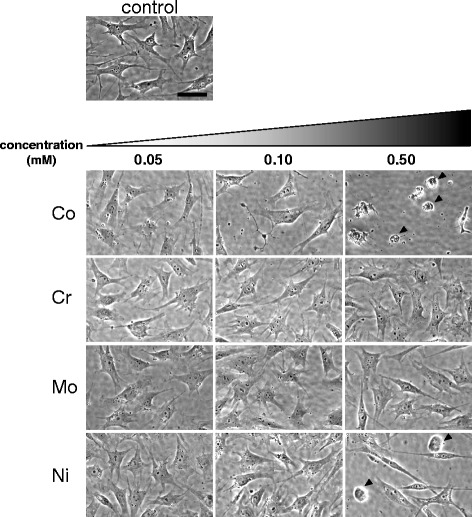
**Phase microscopic observation of MLO-Y4 osteocytes after metal ion treatment for 24 hr.** Round-shaped cells that lost long dendritic cell processes characteristic to MLO-Y4 osteocytes were observed in both Co- and Ni-ion-treated cells at 0.50 mM (arrowheads). Scale bar =50 μm.

The MLO-Y4 cells treated with Cr and Mo ion solution did not reveal any specific changes under phase-contrast microscopy at any concentration. While the cells in the control group showed cell confluence, cells treated with Co at a concentration above 0.10 mM and Ni ion at 0.50 mM revealed a decrease in the number of adherent MLO-Y4 cells. In addition, MLO-Y4 osteocytes adopted a round shape morphology in sharp contrast to their usual long dendritic cell processes after treatment with both Co and Ni ions at 0.50 mM (Figure 3; arrowheads).

**Figure 4:**
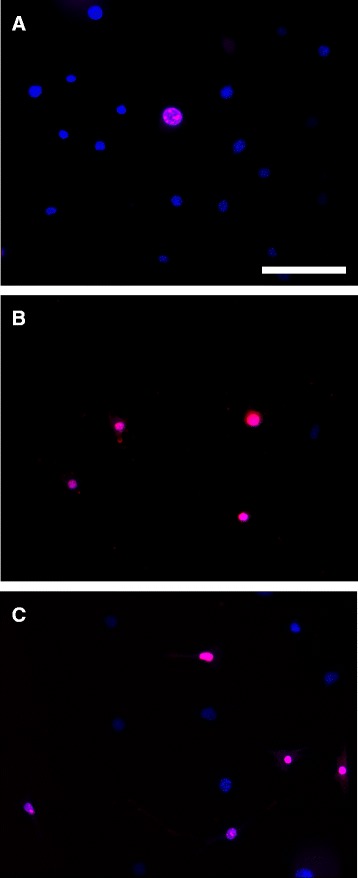
**Fluorescent microscopic observation of MLO-Y4 osteocytes.** Fluorescent microscopic observation of MLO-Y4 osteocytes after **(A)** 0.00 mM (control) or 0.50 mM **(B)** Co and **(C)** Ni ion solution treatment for 24 hr. Total cell numbers and ratio of dead cells were assessed following Hoechst 33342 and propidium iodine staining. Scale bar =100 μm.

For Co and Ni ions, fluorescent microscopic analysis was performed for 0.50-mM treated samples. The fluorescent image showed a decrease in cell numbers as depicted by blue Hoechst 33342 positive cells, compared to controls. Moreover, there was a higher ratio of red propidium iodine positive cells over total cells at 0.50 mM of Co (Figure 4B) and Ni (Figure 4C), compared to control (Figure 4A).

## Discussion

The objective of this study was to determine the osteocyte response to soluble metal ions that are reported to be released from metal-on-metal (Co-Cr-Mo) implants [5–8]. For this, we used the MLO-Y4 cells, an osteocyte cell line, which shows numerous osteocytic characteristics [23]. Utilizing this cell line has the advantage of providing a uniform population of well-characterized cells, unlike bone-derived primary cells, which contain a number of different cell types.

The present study demonstrated that particular metal ions that are reported to be released from a metal-on-metal bearing surface [5–8] have effects on osteocytes that are metal-specific. Co and Ni ions, demonstrated a cytotoxic effect on MLO-Y4 osteocytes while Cr and Mo ions had a proliferative effect. This is the first report to show that metal ion solutions can have cytotoxic effects on osteocytes *in vitro*. Among all metal ion solutions studied, Co and Ni ions had a significant cytotoxic effect on MLO-Y4 cells at concentrations above 0.05 mM and at 0.10 mM, respectively, in a dose-dependent manner. The results from the cell death ELISA demonstrate that Co ion exposure induced osteocyte necrosis rather than apoptosis. On the other hand, Ni ion exposure induced osteocytes to apoptosis. Morphological assays support the results from the cytotoxic assays by showing fewer cells overall and cells with rounded morphology when treated with Co and Ni ions at high concentration (0.50 mM). Additionally, fluorescent images provide qualitative evidence of a decrease in total cell number and higher numbers of dead osteocytes treated with Co and Ni ions at high concentration (0.50 mM).

Our findings are in agreement with previous studies that showed the negative effects of metal ions using different cell types, such as fibroblasts, osteoblasts, lymphocytes, and macrophages [21,22,24–31]. Caicedo et al. reported that Ni ions induced T cell apoptosis at 0.10 mM and Co ion was the most toxic metal, reducing the T cell viability at 0.50 mM. Whereas the negative effects of Cr and Mo were mostly induced at the higher concentration (5.00 mM) [21]. Similarly, Hallab et al. demonstrated cytotoxic effects of Co and Ni ions on osteoblasts, fibroblasts, and lymphocytes at a low dose (0.01 to 2.00 mM), whereas the same level of cytotoxicity from Cr and Mo ions on each cell types were achieved at approximately 10-fold higher dose (1.00 to >10.00 mM) [22]. Several other studies also reported Co ion as more toxic for the induction of osteoblast and macrophage cell death compared to Cr ion [24–27].

It has been reported that osteocytes exist near the implant surface, *in vivo* [32,33]. A study using conventional and high-voltage transmission electron microscopy demonstrated that osteocytes were routinely observed at the bone-implant surface and extend cellular processes directly onto the implant surface through their canaliculi [32]. However, the clinical relevance of metal-ion-induced osteocyte toxicity *in vitro* remains uncertain. Information of the concentration of metal ions in periprosthetic bone is limited when compared to other human body fluids and tissues. Busse et al. successfully detected a considerable amount of heavy metal in the periprosthetic mineralized bone tissue [20]. By utilizing proton-induced X-ray emission microanalysis, Co concentration in the periprosthetic mineralized bone tissue (Gruen zone 7) was 38 to 413 parts per million (ppm) [20], although no distinction was made between entrapped particles and homogeneously distributed ions in the bone matrix. Converting ppm concentrations to mM [28]: ppm/molecular weight (Co: 58.93) = mM, the concentration of Co in the bone matrix will be greater than 0.64 mM. This information indicates, at least for Co, that the cytotoxic effect in osteocytes in the current study which was observed beyond 0.10 mM may be clinically relevant.

Our current findings may have important implications regarding the potential role of osteocytes in periprosthetic bone resorption, since several studies have shown that osteocyte apoptosis contributes to the control of local bone resorption by sending signals to recruit osteoclasts. Osteocytes, historically, have been considered to be metabolically inactive cells [34] with little attention given to them, compared with studies on osteoblasts and osteoclasts [35]. However, there is now evidence that osteocytes regulate bone formation and resorption [12–16]. Several studies have demonstrated that bone fatigue-induced microdamages or unloading leads osteocytes to undergo apoptosis and the involved bone segment is subsequently resorbed by osteoclasts [12–15]. Cardoso et al. demonstrated that rats treated with the pan-caspase inhibitor, an apoptosis inhibitor, completely blocked both fatigue-induced osteocyte apoptosis and the activation of bone resorption by osteoclasts, suggesting that apoptotic events following a fatigue-induced microdamage may play a substantial role in determining the course of bone remodeling [13]. In addition, Tatsumi et al. generated osteocyte-ablated transgenic mice and demonstrated significantly lower bone strength with increased intracortical porosity and microfractures, osteoblastic dysfunction, and trabecular bone loss with microstructural deterioration and adipose tissue proliferation in the bone marrow space [14]. These results support previous findings in which osteocyte apoptosis contributes to the control of local bone resorption by sending signals to recruit osteoclasts. Our data support the concept that factors other than excessive loading or unloading may induce osteocytes to undergo apoptosis and activate bone resorption.

The current study has several limitations that will require further research. Although, it has been reported that osteocytes exist near the implant surface [32,33] and that considerable amount (0.64 to 7.00 mM) of heavy metal (Co) was detected in the periprosthetic mineralized bone tissue [20], the exact concentration of the metal ions which osteocytes experience *in vivo* remains unknown. The concentrations of the metal ions in this study were decided by an assumption that the matrix-embedded osteocytes experience the amount of metals that were detected in the bone matrix. Further experiments should be conducted to elucidate the concentration of metal ions which osteocytes are exposed *in vivo*. Moreover, currently, direct evidence showing the ability of metal ions released from metal implants to induce osteocyte apoptosis and trigger bone remodeling *in vivo* is not available. However, it has been reported that osteocyte apoptosis induced by weightlessness or a bone fatigue microdamage triggers bone remodeling *in vivo* [12,13]. Therefore, evidence whether osteocyte apoptosis induced by metal ions stimulate bone remodeling and their capability to initiate subsequent implant loosening awaits future study. Another related limitation in this type of experiment involves use of cell lines as an approximation of osteocytes *in vivo*. For this, at this stage, our preliminary findings are only able to propose osteocytes as a possible cell type that may contribute to implant failure. Lastly, the mechanism is unknown for how osteocytes recognize/sense the metal ions. To date, whether cytotoxic effects of Co and Ni, observed in our study, require intracellular uptake of these metal ions or are mediated via surface-based receptors or a destabilization of the cell membrane integrity is unknown. This remains to be investigated.

## Conclusions

In conclusion, we have demonstrated that metal ions that are reported to be released from metal-on-metal bearing surfaces [5–8] have potentially cytotoxic effects on osteocytes. These effects together with recent knowledge on the connection between osteocyte apoptosis and initiation of bone resorption suggest that osteocytes may play an important role in regulating periprosthetic bone resorption following total hip arthroplasty by becoming apoptotic upon metal challenge. These findings warrant further *in vivo* study of the degree of osteocyte involvement in the pathogenesis of aseptic loosening.
